# Endoscopic resection with adjuvant treatment versus esophagectomy for early-stage esophageal cancer

**DOI:** 10.1007/s00464-021-08466-2

**Published:** 2021-04-23

**Authors:** Binhao Huang, Maria Christine Xu, Arjun Pennathur, Zhigang Li, Zhiguo Liu, Qi Wu, Jing Wang, Kongjia Luo, Jianying Bai, Zhi Wei, Jiaqing Xiang, Wentao Fang, Jie Zhang

**Affiliations:** 1 Department of Thoracic Surgery, Shanghai Chest Hospital, Shanghai, China; 2 Department of Gastric Surgery, Fudan University Shanghai Cancer Center, Shanghai, China; 3 Department of Oncology, Shanghai Medical College, Fudan University, Shanghai, China; 4 Department of Cardiothoracic Surgery, University of Pittsburgh Medical Center, Pittsburgh, PA USA; 5 Xijing Hospital of Digestive Disease, The Air Force Medical University, Xian, China; 6 Endoscopy Center, Peking University Cancer Hospital, Beijing, China; 7 State Key Laboratory of Oncology in South China, Collaborative Innovation Center for Cancer Medicine, Sun Yat-Sen University Cancer Center, Guangzhou, China; 8 Guangdong Esophageal Cancer Institute (GECI), Guangzhou, China; 9 Department of Gastroenterology. Xinqiao Hospital, Third Military Medical University, Chongqing, China; 10 Gastroenterology Department, The 960th Hospital of the PLA, Jinan, China; 11 University of Toronto Schools, Toronto, Canada; 12 Department of Thoracic Surgery, Fudan University Shanghai Cancer Center, Shanghai, China

**Keywords:** Esophageal neoplasms, Endoscopic resection, Chemoradiotherapy, Esophagectomy

## Abstract

**Objective:**

To evaluate the outcome following the strategy of endoscopic R0 resection (ER) plus adjuvant treatment (AT) versus esophagectomy for esophageal squamous cell cancer in T1a invading muscularis mucosa (M3)-T1b stage.

**Methods:**

We evaluated the outcomes of 46 esophageal squamous cell cancer (ESCC) patients with T1aM3-T1b stage who underwent ER + AT from the Esophageal Cancer Endoscopic Therapy Consortium (ECETC) and compared these outcomes to 92 patients who underwent esophagectomy. Propensity score matching (1:2) was used, with overall survival (OS) and relapse-free survival (RFS) being compared between the two groups.

**Results:**

During a median follow-up of 32 months, there were no statistical differences (*P* = 0.226) in OS between the two groups. The 1-, 2-, and 3-year overall survival in the esophagectomy group was 95%, 91%, and 84%, respectively. There were no mortalities within three years in the ER + AT group. The RFS between the two groups was also not significantly different (*P* = 0.938). The 1-, 2-, and 3-year RFS of patients in the esophagectomy group was 90%, 90%, and 83%, respectively, while it was 97%, 94%, and 74% in the ER + AT group, respectively. The local recurrence rates between the two groups were not significantly different (*P* = 0.277).

**Conclusions:**

This first multicenter analysis showed similar outcomes were found regarding OS and RFS between the two groups in T1aM3-T1b stage patients. ER + AT may be considered in high-risk patients or for those who refuse esophagectomy.

**Supplementary Information:**

The online version contains supplementary material available at 10.1007/s00464-021-08466-2.

Esophageal carcinoma has become one of the leading causes of cancer death worldwide [1]. In China, it is still ranked among the top four malignant tumors for its high morbidity and tumor-related mortality across the country [2]. Therefore, detecting esophageal cancer in the early stage and finding an appropriate therapeutic intervention is urgently needed for a better prognosis.

The past decade has witnessed a boom in endoscopy. It has been widely applied in the screening, diagnosis, and therapy of superficial esophageal cancer owing to its minimally invasive approach [3]. Endoscopic resection (ER) is recommended for selected patients with low-risk characteristics who have early-stage cancer without evidence of lymph node metastasis. For tumors within lamina propria (T1am1-m2), endoscopic R0 resection is believed to be sufficient for a good prognosis [4]. However, the risk of lymph node metastasis increases rapidly when tumors invade muscularis mucosae (T1am3, 9%), and the risks are even higher when invading beyond submucosa (16%, 38%, and 64% for T1bsm1, sm2, and sm3, respectively) [5]. So, ER alone is considered controversial as a curative treatment for these patients.

A Japanese clinical trial (JCOG9708) found that patients with T1 esophageal squamous cell carcinoma (ESCC) who underwent definitive chemoradiotherapy would have a comparable survival rate to those after surgery [6]. Potential metastatic lymph nodes could be covered by chemoradiation, but the local recurrence rate was a significant problem because the primary lesion was not removed by chemoradiotherapy [7]. Furthermore, invasion depth could not be precisely evaluated even though endoscopic ultrasonography (EUS) was scheduled before therapy [8]. In other words, some patients might have undergone overtreatment or undertreatment. It was believed that ER provided a more accurate depth of invasion information than EUS [9, 10]. Therefore, definite chemoradiotherapy without ER is insufficient for these patients either.

Esophagectomy has been considered a standard treatment for those with a high risk of lymph node metastasis. Compared to traditional surgeries like esophagectomy, ER has the advantages of fewer postoperative complications and decreased mortality [11, 12]. However, lymphadenectomy is not performed during the ER procedure. Hence, some investigators have suggested the addition of chemoradiotherapy to ER (ER + AT) instead of an esophagectomy [13–17] to address the potential lymph node metastasis, with the hope that this strategy can benefit from the combined advantages of ER + AT while ameliorating their shortcomings. Until now, only small single-arm and single-center studies exist [18], and no studies have focused on evaluating this strategy in T1 am3-T1b stage esophageal cancer.

This first multicenter study was conducted to evaluate the outcomes following the strategy of ER + AT for esophageal cancer in the T1aM3-T1b stage versus esophagectomy. This study was based on data from the Esophageal Cancer Endoscopic Therapy Consortium (ECETC).

## Methods

### Data collection

The multicenter data were collected from the ECETC, which consisted of information from 9 independent hospitals including the University of Pittsburgh Medical Center, Shanghai Chest Hospital, Fudan University Shanghai Cancer Center, Xijing Hospital of Digestive Disease, Peking University Cancer Hospital, Sun Yat-Sen University Cancer Center, Xinqiao Hospital, The 960th Hospital of the PLA, and Wuhan General Hospital of the Guangzhou Military. Patient data between September 2011 and February 2017 were collected. The inclusion criteria for patient data were as follows: (1) cN0 and cM0, (2) endoscopic R0 resection, (3) pathologically diagnosed as T1aM3-T1b esophageal squamous cell cancer, and (4) treated with additional chemotherapy or/and radiotherapy after endoscopic resection. Forty-six patients with esophageal cancer in early stages were included in this study as the ER + AT group. Patients who underwent esophagectomy from the main center were chosen as the control group for the comparison. All patients provided written informed consent before treatment. They were also informed that the data would be for research use and might be published after de-identification. This study was approved by the Committee for Ethical Review of Research (Review Board No. 090977-1).

### ER techniques

Endoscopic resection of ECETC included endoscopic multi-band mucosectomy (EMBM) and endoscopic submucosal dissection (ESD). EMBM was performed using the Duette Multiband Mucosectomy Kit (DT-6, Cook Medical, Bloomington, IN, USA). The T-knife, dual knife, or VS knife (Erbe Elektromedizin, GmbH, Tübingen, Germany) was used for ESD, under the ENDO CUT IQ model (Erbe platform system). We recommend endoscopic surveillance at 1, 3, 6, 12, 18, and 24 months within the first 2 years, after curative ER for superficial esophageal cancer.

### Chemo/radiotherapy

The patients who underwent ER received postoperative treatment. The treatments were determined after discussion between doctors and patients. ER patients were not offered esophagectomy in these conditions: (1) patients performance status did not allow the surgery; (2) patients strongly desired organ preservation; (3) patients were likely to develop postoperative complications; (4) presence of other relevant social factors, such as a lack of insurance to cover esophagectomy. The primary and standard plan of the chemotherapy regimen involved intravenous infusion of cisplatin (70 mg/m^2^/day) on days 1 and 5-fluorouracil (5-FU) (700 mg/m^2^/day for 24 h) on days 1 to 5, every 4 weeks. A total dose of 61.2 Gy radiation in 34 fractions (5 days per week at 1.8 Gy/day) was applied. The clinical target volume included the pre-therapeutic extension of the primary tumor and regional lymph nodes with some modification in different centers. Reginal nodes involved in radiation are based on tumor location. Supra-clavicular, upper mediastinal, and subcarinal nodal areas are for the upper thoracic esophagus; mediastinal and perigastric nodal areas are for the middle thoracic esophagus; and mediastinal, perigastric, and celiac nodal areas are for the lower thoracic esophagus.

### Definitive surgery

Esophagectomy and lymphadenectomy were performed in the main center, and the surgical procedure was determined based on the feature of the lesion and the experience of the primary surgeon. The surgery was conducted by Ivor Lewis and three-incision/McKeown operative approach. Patients had a follow-up visit in the clinic after esophagectomy every 3 months in the first year, every 4–6 months in the second year, and annually thereafter. For the esophagectomy group, yearly endoscopic surveillance was recommended. Patients would receive additional endoscopic examination if they had dysphagia. Computed tomography (CT) imaging was performed every 3 months within the first 2 years, and every half year afterward.

### Pathological diagnosis and staging

Tumor location was determined by a report of endoscopy before surgery. Tumor stages were reassessed based on the 2017 International Union Against Cancer (UICC) and the American Joint Committee on Cancer (AJCC) 8th version TNM classification system. The depth of invasion, surgical margin, and lymphovascular invasion (LVI) were examined and determined by at least two independent pathologists who were blinded to clinical information.

### Propensity score matching (PSM)

Propensity score matching [19, 20] was performed with 1:2 nearest-neighbor matching without replacement to identify matched cohorts for the two treatment modalities (ER + AT and esophagectomy only). This method was adopted to balance the covariates, which could have influenced the survival outcomes between the two groups.

In the ECETC set, we included gender, age, location, depth of invasion, and LVI for matching. Based on this set of covariates, the propensity score was estimated using logistic regression. Standardized mean differences (SMD) were used to assess the balance of baseline data [21]. The statistical analyses were conducted using R version 3.3.1 software (R, CA, USA).

### Statistical analysis

The number and percentage of patients for each subgroup were listed unless otherwise specified. Local recurrence included the recurrence at the primary site, mediastinal lymph nodes and a metachronous lesion. Pearson’s *χ*^2^ test or Fisher’s exact test was used for comparing frequencies for categorical variables, and one-way analysis of variance (ANOVA) or Student’s *t*-test was used for continuity variables. Kaplan–Meier curve and the log-rank test were used to evaluate overall survival (OS) and relapse-free survival (RFS). Competing risk method was used to evaluate local recurrence rate with death without local recurrence treated as competing risk event. The Cox regression model was used for univariate and multivariate analysis regarding OS and RFS. All statistical analyses were performed in SPSS version 22.0, and figures were generated by Prism version 6.0 and R version 3.3.1 software (R, CA, USA). A *P* value of < 0.05 was considered statistically significant for all analyses.

## Results

### Characteristics of the patients before matching

Data of the patients from the ECETC set before and after PSM are listed in Table 1. After evaluation by pathologists, the patients were all diagnosed with ESCC. Before matching, there were more patients with lower esophageal cancer (*P* < 0.001, SMD = 0.850) and T1b (*P* = 0.041, SMD = 0.313) in the esophagectomy group. According to the pathologic information after esophagectomy, 15% (47/311) of patients had positive lymph nodes and pathologic upstage. The percentage of upstaging was 4.5% and 19.7% for T1a and T1b patients, respectively.

**Table 1:** Distribution of variables between ER + AT and esophagectomy groups before and after propensity score matching in the ECETC Set

	Before matching		*P* value	SMD (95%CI)	After matching		*P* value	SMD (95%CI)
	ER + AT	Esophagectomy			ER + AT	Esophagectomy		
	*N* = 46	*N* = 311			*N* = 46	*N* = 92		
Gender			0.771	0.045 (− 0.264 to 0.355)			0.672	0.076 (− 0.278 to 0.430)
Male	34 (73.91%)	236 (75.88%)			34 (73.91%)	71 (77.17%)		
Female	12 (26.09%)	75 (24.12%)			12 (26.09%)	21 (22.83%)		
Age, years (${\bar{\text x}} \pm {\text{s}}$)	61.13 ± 8.10	60.84 ± 7.78	0.811	0.165 (− 0.145 to 0.474)	61.13 ± 8.10	61.07 ± 6.72	0.960	0.009 (− 0.345 to 0.363)
Position			< 0.001*	0.850 (0.534–1.166)			0.739	0.128 (− 0.226 to 0.482)
Upper	13 (28.26%)	30 (9.65%)			13 (28.26%)	21 (22.83%)		
Middle	27 (58.70%)	135 (43.41%)			27 (58.70%)	60 (65.22%)		
Lower	6 (13.04%)	146 (46.95%)			6 (13.04%)	11 (11.96%)		
Stage			0.041*	0.313 (0.003–0.624)			0.386	0.156 (− 0.198 to 0.510)
T1a	20 (43.48%)	89 (28.62%)			20 (43.48%)	33 (35.87%)		
T1b	26 (56.52%)	222 (71.38%)			26 (56.52%)	59 (64.13%)		
LVI			0.659	0.109 (− 0.201 to 0.418)			1.000	0.036 (− 0.318 to 0.390)
Negative	41 (89.13%)	287 (92.28%)			41 (89.13%)	83 (90.22%)		
Positive	5 (10.87%)	24 (7.72%)			5 (10.87%)	9 (9.78%)		

Data presented as *N* (%) unless otherwise specified

*ECETC* esophageal cancer endoscopic therapy consortium, *ER* endoscopic resection, *AT* adjuvant therapy, *SMD* standardized mean difference

*P* value was derived from *χ*^2^ test or Fisher’s exact test for categorical variables, and ANOVA for continuous variables

### Characteristics of the patients after matching

After matching, all the factors were balanced, and we finally included 46 patients in the ER + AT group with 34 males and 12 females, with an age range of 61.13 ± 8.10 years. Overall, 74% received additional radiotherapy, while 26% received chemotherapy in the ER + AT group. Regarding tumor stage, 20 patients were T1am3, 3 patients were T1bsm1, and 17 were ≥ T1bsm2. It is not routine for all centers to distinguish between sm1, sm2, and sm3 within T1b tumors. Another 6 tumors that invaded submucosa were classified into T1bsmx.

There were 92 patients in the esophagectomy group with 71 males and 21 females, with an age range of 61.07 ± 6.72 years. No difference was found in gender, age, location, T stage, and LVI between the two groups (*P* > 0.05, SMD < 0.200). The ER + AT group had a longer tumor length (median, 4 cm vs 2 cm; *P* < 0.001) compared to esophagectomy. We did not include this for PSM because patients could not be fully matched. Instead, we performed multivariate analysis, including tumor length as a covariate. After matching, the percentage of lymph node upstaging was 9.8% (9/92).

### Comparison of overall survival

**Fig. 1:**
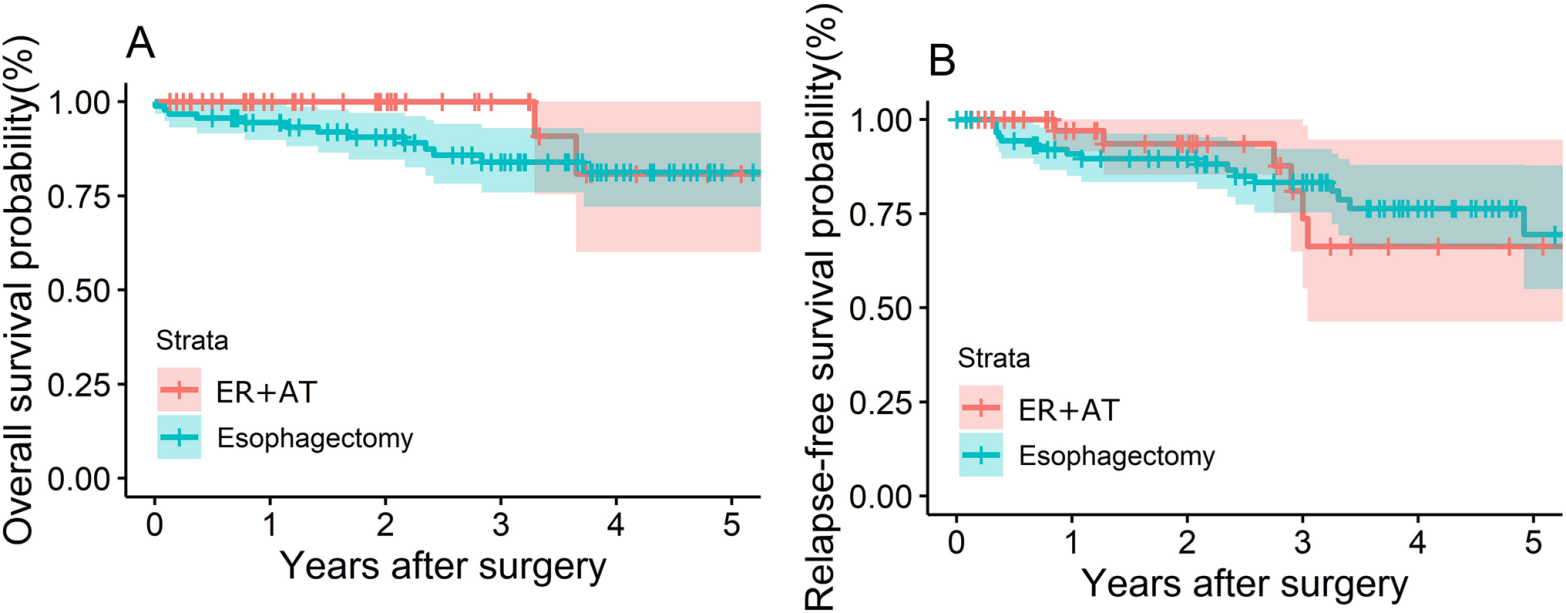
Survival analysis by different therapeutic strategy in the ECETC set. **A** KM curves for overall survival with abstracted number at risk displayed (HR 2.43 with 95%CI 0.78 to 7.56, *P* = 0.226). **B** KM curves for relapse-free survival with abstracted number at risk displayed (HR 1.04, 95% CI 0.41 to 2.60, *P* = 0.938)

There were no statistical differences (HR 2.43 with 95%CI 0.78 to 7.56, *P* = 0.226) in OS between the two groups (Fig. 1A). After a median follow-up of 38 months, one-, two-, and three-year OS in the esophagectomy group was 95%, 91%, and 84%, respectively. Specifically, there were 10 cancer-specific deaths in the esophagectomy group, and 2 perioperative deaths and 1 non-tumor death were excluded from this number. There were no mortalities within 3 years in the ER + AT group, with a median follow-up of 30 months. There were 2 cancer-specific deaths in the ER + AT group after 3 years.

### Comparison of relapse-free survival and local recurrence rate

The RFS between the two groups was not significantly different (HR = 1.04, 95% CI 0.41 to 2.60, *P* = 0.938) (Fig. 1B). The 1-, 2-, and 3-year RFS rate of patients in the esophagectomy group was 90%, 90%, and 83%, respectively, while it was 97%, 94%, and 74% in the ER + AT group, respectively. Local recurrence included the recurrence at the primary site, mediastinal lymph nodes and a metachronous lesion. The local recurrence rates between the two groups were not significantly different (HR 0.50, 95%CI 0.12 to 2.11, *P* = 0.277) (Supplementary Fig. 1).

In ER + AT group, there was no difference in overall survival (Supplementary Fig. 2A), relapse-free survival (Supplementary Fig. 2B) or local recurrence rate (Supplementary Fig. 2C) between those who received chemotherapy and radiation (*P* = 0.053, 0.300 and 0.501, respectively).

### Further analysis in Cox regression models

To strengthen our findings, we also conducted univariate and multivariate analysis using Cox regression models and included those factors which may influence prognosis. Table 2 presents the results of the comparisons OS and RFS between the study groups. Compared to esophagectomy, ER + AT had a similar OS (*P* = 0.241) and RFS (*P* = 0.938) in the univariate model. A further multivariate analysis that included all the clinicopathologic information validated the similarity of OS (*P* = 0.158) and RFS (*P* = 0.681) between ER + AT and esophagectomy. A sensitivity analysis using the before matching cohort to conduct multivariable regression model also found comparable OS (*P* = 0.180) and RFS (*P* = 0.884) (Table 3).

**Table 2:** Cox regression model for comparison between ER + AT and esophagectomy

ER + AT vs Esophagectomy	Overall survival		Relapse-free survival	
	Hazard ratio (95% CI)	*P* value	Hazard ratio (95% CI)	*P* value
Univariate model	0.409 (0.092–1.822)	0.241	0.964 (0.380–2.446)	0.938
Multivariate model	0.299 (0.056–1.596)	0.158	1.272 (0.404–4.005)	0.681

Multivariate model: Including gender, age, tumor location, tumor length, stage and LVI as covariates

*ECETC* esophageal cancer endoscopic therapy consortium, *ER* endoscopic resection, *AT* adjuvant therapy

**Table 3:** Multivariable Cox regression model for comparison between ER + AT and esophagectomy using the before matching cohort

Overall survival		Relapse-free survival	
Hazard ratio (95% CI)	*P* value	Hazard ratio (95% CI)	*P* value
0.373 (0.088–1.587)	0.180	0.934 (0.386–2.273)	0.884

The adjusted covariates include gender, age, tumor location, tumor length, stage and LVI

## Discussion

Endoscopic resection alone might not be enough for patients with T1am3-T1b esophageal cancer mainly due to inadequate resection and unremoved positive lymph nodes, which are significantly related to more reduced survival. Our preliminary data also demonstrated Tm3/ sm1 patient who underwent endoscopic resection alone had worse survival compared with esophagectomy. Therefore, investigators have been trying for years to find the risk factors that can predict lymph node metastasis [22–25], and those were found to predict high risk are recommended for an indication of esophagectomy. A scoring system based on the National Cancer Database indicated that layer of invasion, tumor size, differentiation, and lymphovascular invasion (LVI) were associated with positive lymph nodes [22]. Sgourakis et al. concluded that the best predictors were sm3 invasion and presence of LVI [26], while Cen et al. also demonstrated that LVI was an independent risk factor for predicting lymph node metastasis [27]. Furthermore, our previous study revealed that LVI had a solid predictive function of lymph node status in multivariate analysis [28]. So, in addition to routine clinicopathologic factors, we also included LVI as one of the variables for PSM.

Here, we introduce an “all-covered mode” as ER + AT strategy, and it can be applied for T1am3-T1b stage esophageal cancer. It was reported as an effective and safe approach in single-center study, and it improved the local control rate [15, 16].The rationale of ER plus adjuvant therapy is that ER can obtain accurate T staging while the primary lesion is removed, and adjuvant therapy can further reduce the potential of both local and mediastinal lymph node recurrence. Moreover, the combination approach preserves the function of the esophagus, which significantly improves the quality of life. Up to now, this is the first multicenter analysis, comparing endoscopic resection with adjuvant treatment versus esophagectomy for early-stage esophageal cancer. Some literatures showed that squamous cell carcinoma of proximal esophagus equivalent survival with CRT vs surgery [29], even in later stages. Kato et al. reported the 87.5% (63 of 72) CR rate of definitive CRT for clinical stage I ESCC, and achieved comparable survival rate to those receiving surgery [6]. Here, we introduced ER before chemo/radiotherapy to obtain an accurate T stage information. Additionally, ER + AT can be applied to middle and distal esophagus cancer too.

In our study, we analyzed the outcomes of ESCC treated with ER and AT from the ECETC, which included 9 hospitals and compared the findings to patients who underwent esophagectomy. We did not find significant differences in OS, RFS, and local recurrence rates between the two groups, despite a tendency of a higher local recurrence rate in the ER + AT group. The results of local recurrence were almost consistent with Nelson’s conclusion, which found that esophageal preservation was associated with an increased risk of local recurrence [30]. However, additional therapy was not applied in Nelson’s study. Given that no local recurrence occurred within 2 years in the ER + AT group, it is reasonable to confirm the effectiveness of subsequent AT. Here, we observed a rising local recurrence rate after 3 years. We assumed this result was mainly due to the following 2 reasons. First, for ER + AT patients, adjuvant therapy could effectively reduce the risk of recurrence and prolong the time to disease progression. Therefore, there was no local recurrence cases in first 3 years after treatment. However, the regional lymph nodes were not resected for these patients, thus became a potential risk factor for some local recurrent cases. Second, metachronous lesion in the later is another explanation, which was caused by preserved esophagus during ER. So we recommended regular follow-up for a long time. In addition, it is also important to educate the patients to quit smoking, limit alcohol to avoid cancer recurrence.

Treatment decision-making for patients with multiple medical problems has always been difficult. In addition to obtaining a better long-term survival, for some patients, advanced age or comorbidities also need to be taken into account [31, 32]. To make the best choice for patients, we need to balance the benefits and risks. ER plus adjuvant therapy is an alternative choice for those patients who are at high risk and are not operative candidates.

The present study had some limitations. First, although the data were collected prospectively from an institutional database, selection bias was unavoidable, as it was a retrospective analysis. PSM was performed to normalize the variables between the two groups to reduce bias. However, unmeasured confounders like patient comorbidity before treatment were not included in this study. Generally, serious comorbidity would be a reason to reject esophagectomy. Considering that more ER + AT patients may have preoperative comorbidity, and relatively better outcomes would have been found if we had matched it, it was acceptable to obtain a similar prognosis without these data. Second, additional therapy to endoscopic resection was not uniform among the different centers. If LVI is positive, patients are more likely to have CT. If the patients are T1b sm2 or sm3, they are more likely to have RT. Additionally, different centers also have their preference for treatment modalities. There is no consensus or guideline for additional treatment, and not all patients in the esophagectomy group underwent initial ER. Heterogeneity of the staging procedure prior to treatment also existed, because it is difficult to standardize procedures across institutions. Third, this study examined patients who had squamous cell carcinoma, and further studies are required to evaluate outcomes in esophageal adenocarcinoma. Finally, the follow-up time was not sufficiently long in this study. Further prospective studies with a cohort of patients and longer follow-up are required to evaluate the strategy of endoscopic resection followed by additional chemoradiotherapy for the treatment of esophageal cancer in T1aM3-T1b stage.

In conclusion, we compared the outcomes of chemoradiotherapy following endoscopic R0 resection for esophageal cancer in the T1aM3-T1b stage versus esophagectomy. Comparable OS, RFS, and local recurrence rates were found between the two groups. The ER plus adjuvant therapy strategy may be considered in ESCC patients who have multiple high-risk factors or those who refuse esophagectomy.

## Supplementary Information

Below is the link to the electronic supplementary material.

Supplementary Figure 1. Cumulative local recurrence rate with abstracted number at risk displayed using competing risk method (HR=0.46, 95%CI 0.12 to 1.79, P=0.26). ECETC: Esophageal Cancer Endoscopic Therapy Consortium; ER: endoscopic resection; AT: adjuvant therapy (JPEG 439 kb)

Supplementary Figure 2. Subgroup analysis on the additional treatment. In ER+AT group, there was no difference in overall survival (A), relapse-free survival (B) or local recurrence rate (C) between those who received chemotherapy and radiation (p=0.053, 0.300 and 0.501, respectively) (JPEG 292 kb)

Supplementary file1 (JPEG 302 kb)

Supplementary file1 (JPEG 288 kb)
