# Supplementary figures and images for: Endoscopic resection with adjuvant treatment versus esophagectomy for early-stage esophageal cancer
Source: Surg Endosc. 2021 Apr 23;36(3):1868–75. doi: 10.1007/s00464-021-08466-2 (PMC8979881; doi:10.1007/s00464-021-08466-2)

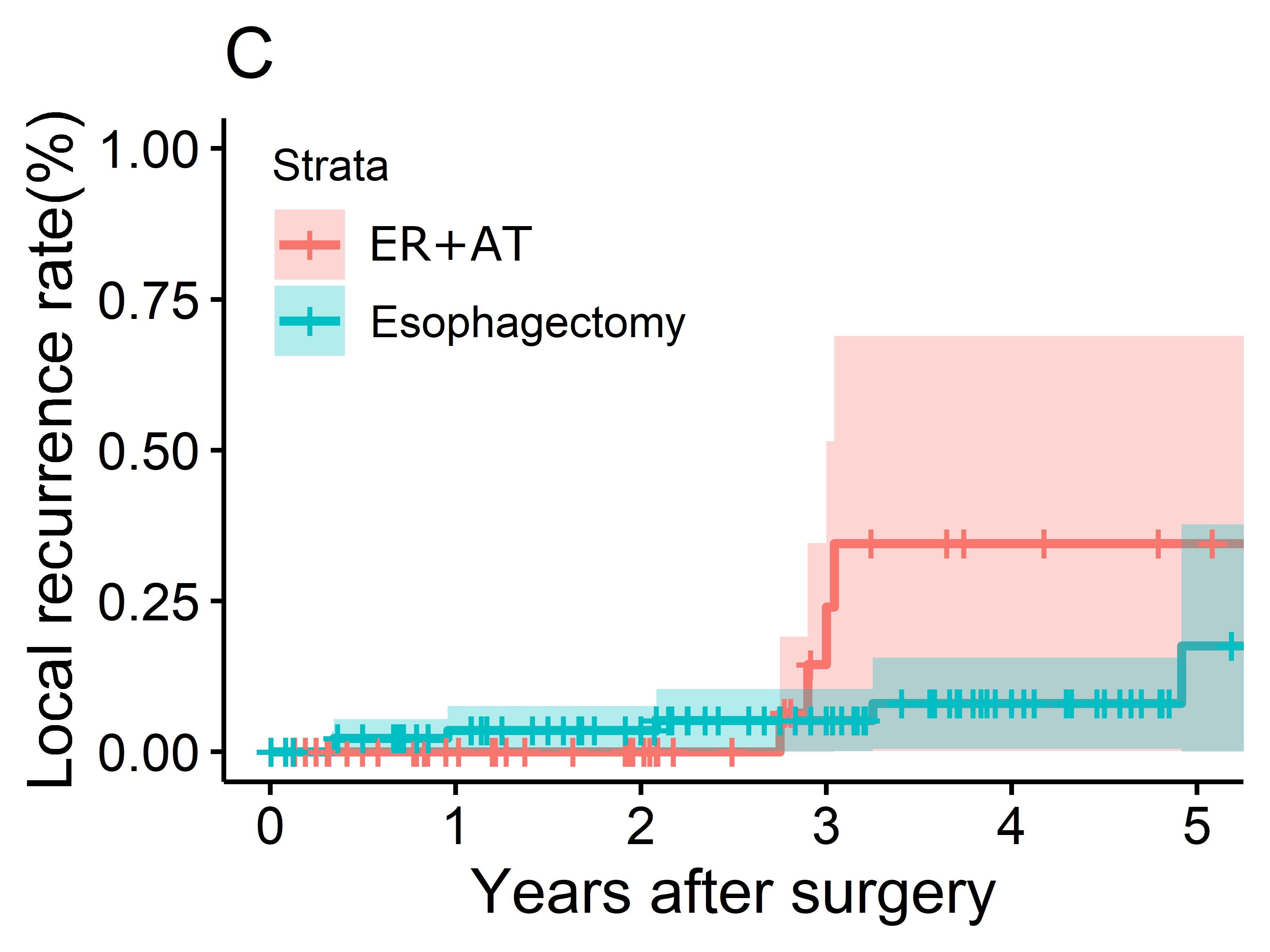

Supplement: Supplementary file 1 — Supplementary Figure 1. Cumulative local recurrence rate with abstracted number at risk displayed using competing risk method (HR=0.46, 95%CI 0.12 to 1.79, P=0.26). ECETC: Esophageal Cancer Endoscopic Therapy Consortium; ER: endoscopic resection; AT: adjuvant therapy (JPEG 439 kb) [file 464_2021_8466_MOESM1_ESM.jpeg]

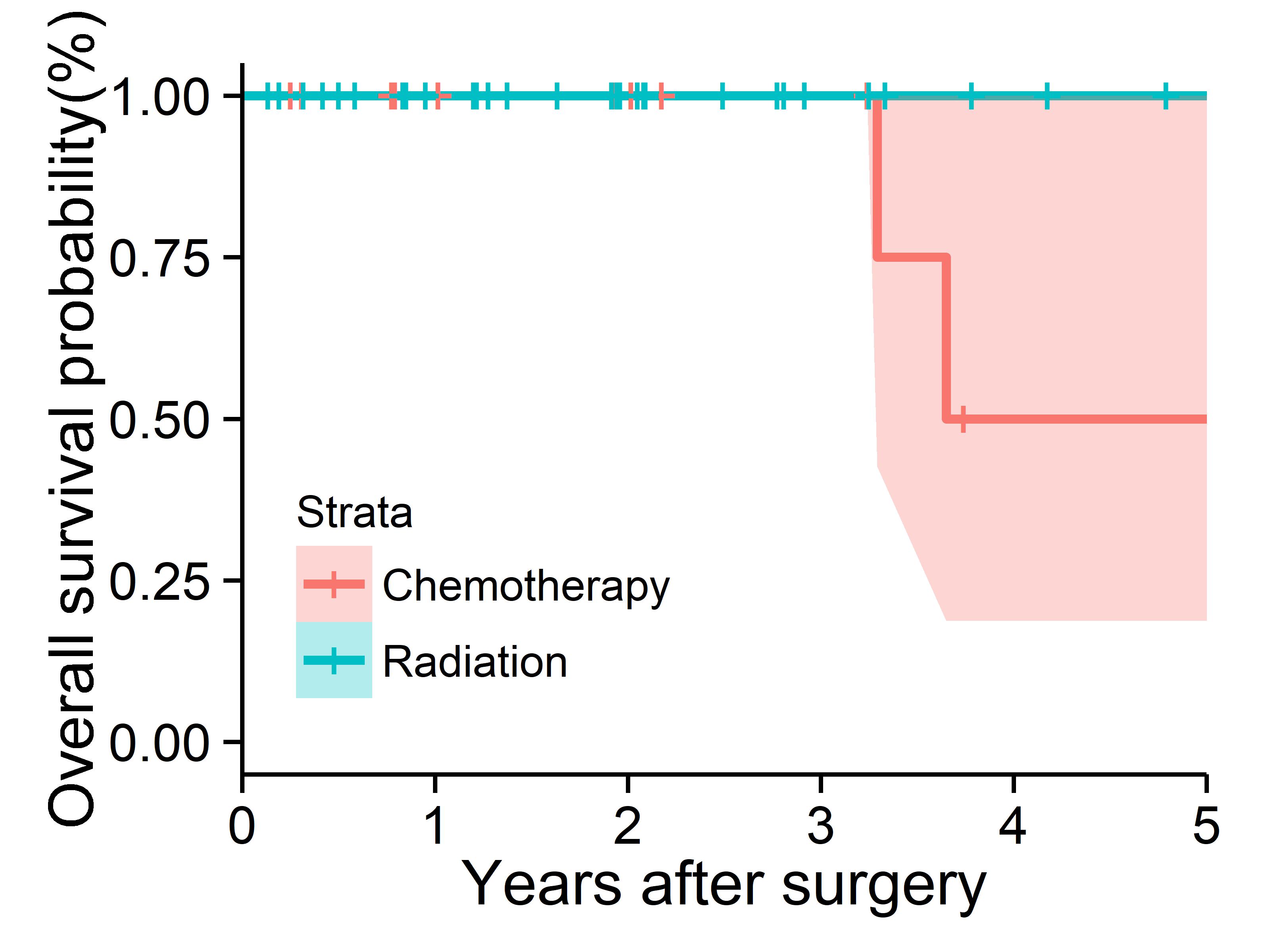

Supplement: Supplementary file 2 — Supplementary Figure 2. Subgroup analysis on the additional treatment. In ER+AT group, there was no difference in overall survival (A), relapse-free survival (B) or local recurrence rate (C) between those who received chemotherapy and radiation (p=0.053, 0.300 and 0.501, respectively) (JPEG 292 kb) [file 464_2021_8466_MOESM2_ESM.jpeg]

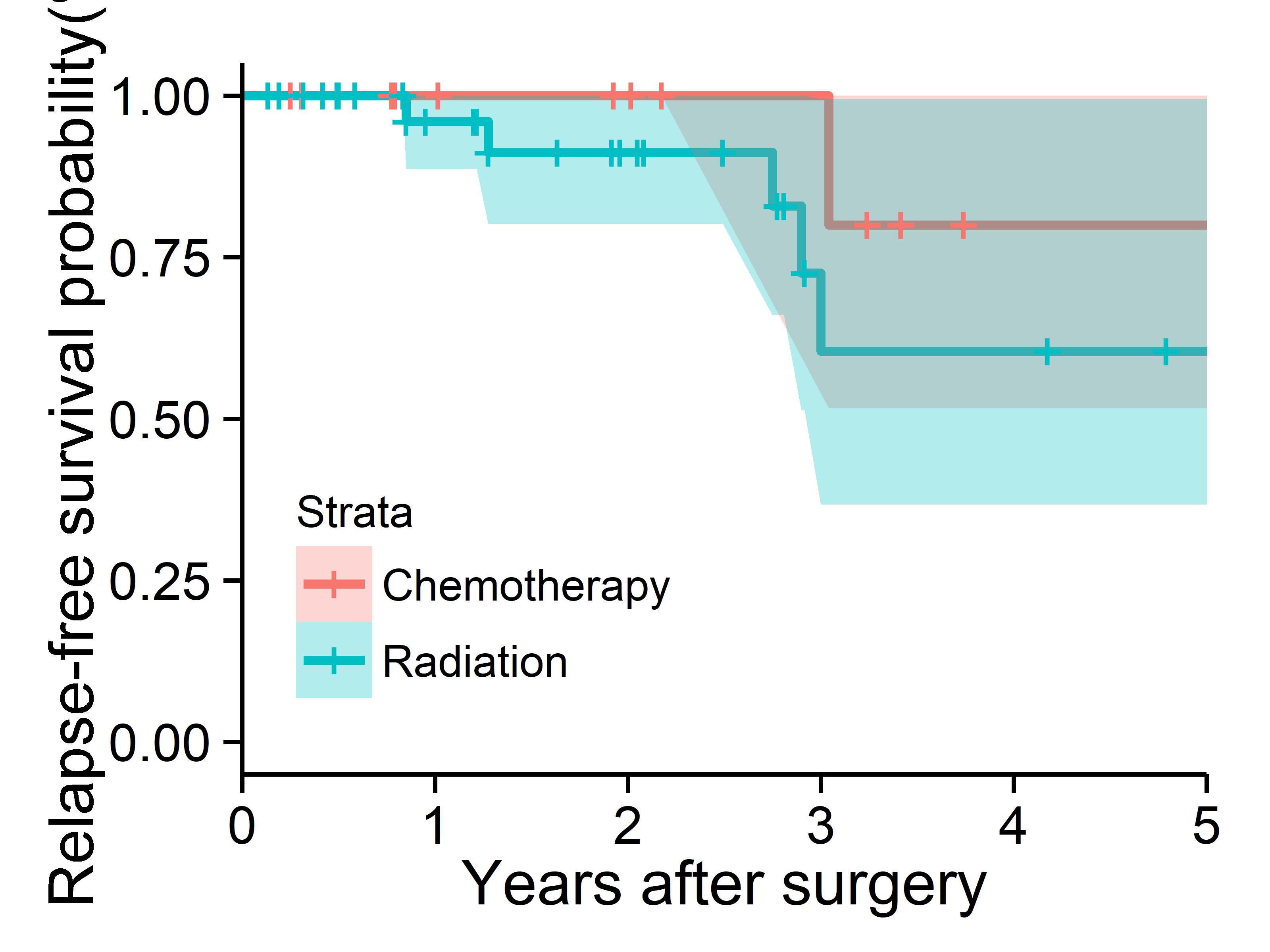

Supplement: Supplementary file 3 — Supplementary file1 (JPEG 302 kb) [file 464_2021_8466_MOESM3_ESM.jpeg]

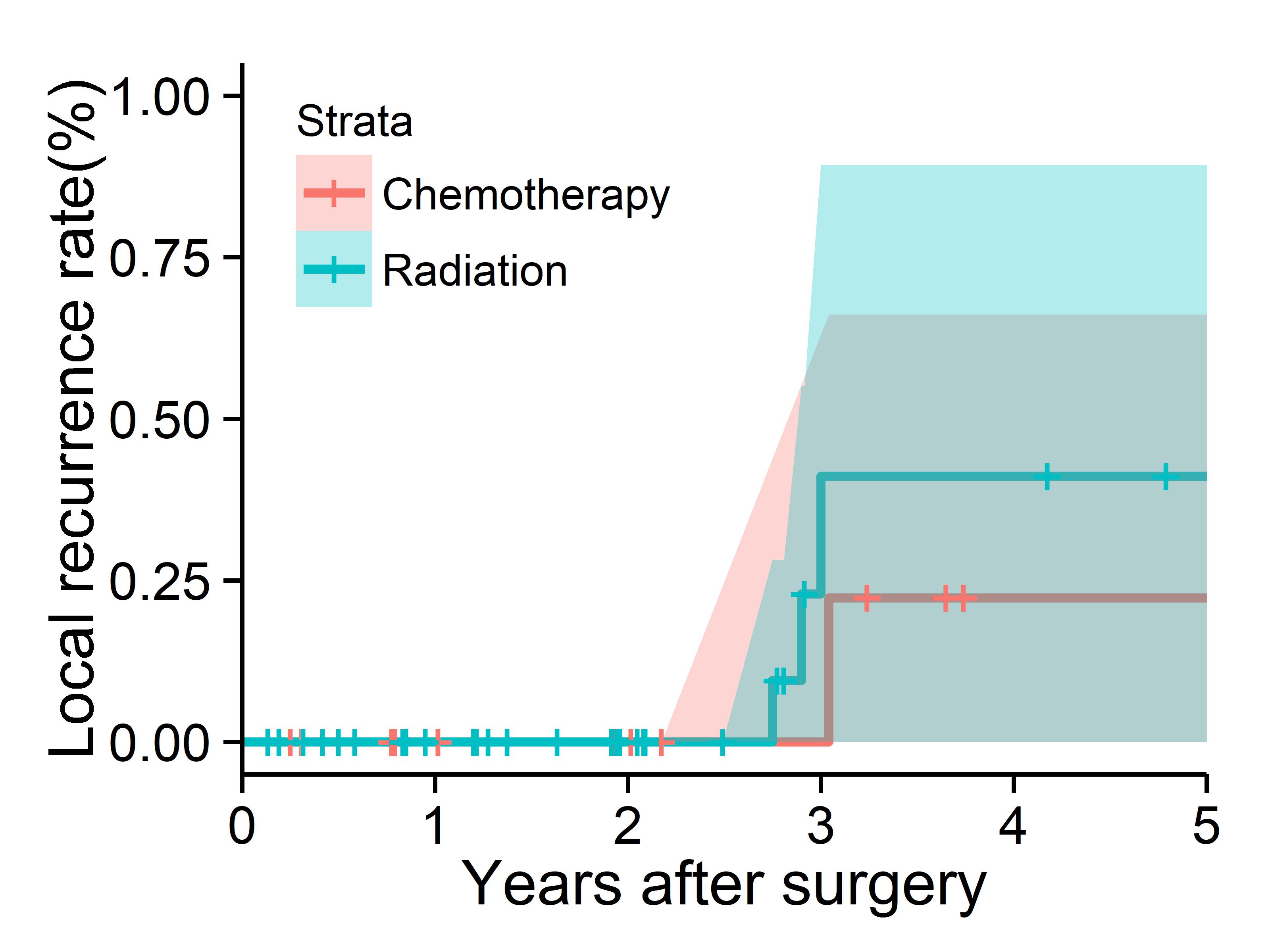

Supplement: Supplementary file 4 — Supplementary file1 (JPEG 288 kb) [file 464_2021_8466_MOESM4_ESM.jpeg]
